# Developing a Stabilizing Formulation of a Live Chimeric Dengue Virus Vaccine Dry Coated on a High-Density Microarray Patch

**DOI:** 10.3390/vaccines9111301

**Published:** 2021-11-09

**Authors:** Jovin J. Y. Choo, Christopher L. D. McMillan, Germain J. P. Fernando, Roy A. Hall, Paul R. Young, Jody Hobson-Peters, David A. Muller

**Affiliations:** 1 Australian Infectious Diseases Research Centre, School of Chemistry and Molecular Biosciences, The University of Queensland, Brisbane, QLD 4072, Australia; j.choo@uq.net.au (J.J.Y.C.); c.mcmillan1@uq.edu.au (C.L.D.M.); g.fernando@uq.edu.au (G.J.P.F.); roy.hall@uq.edu.au (R.A.H.); p.young@uq.edu.au (P.R.Y.); j.peters2@uq.edu.au (J.H.-P.); 2 Translational Research Institute, Vaxxas Pty Ltd., 37 Kent Street, Brisbane, QLD 4102, Australia

**Keywords:** dengue, high-density microarray patch, microneedles, dengue vaccine, chimeric virus, formulation

## Abstract

Alternative delivery systems such as the high-density microarray patch (HD-MAP) are being widely explored due to the variety of benefits they offer over traditional vaccine delivery methods. As vaccines are dry coated onto the HD-MAP, there is a need to ensure the stability of the vaccine in a solid state upon dry down. Other challenges faced are the structural stability during storage as a dried vaccine and during reconstitution upon application into the skin. Using a novel live chimeric virus vaccine candidate, BinJ/DENV2-prME, we explored a panel of pharmaceutical excipients to mitigate vaccine loss during the drying and storage process. This screening identified human serum albumin (HSA) as the lead stabilizing excipient. When bDENV2-coated HD-MAPs were stored at 4 °C for a month, we found complete retention of vaccine potency as assessed by the generation of potent virus-neutralizing antibody responses in mice. We also demonstrated that HD-MAP wear time did not influence vaccine deposition into the skin or the corresponding immunological outcomes. The final candidate formulation with HSA maintained ~100% percentage recovery after 6 months of storage at 4 °C.

## 1. Introduction

Dengue is a systemic viral infection with approximately 390 million infections annually, of which 96 million result in disease of varying severity [1]. Dengue virus (DENV) belongs to the *Flavivirus* genus and is transmitted via the bite of an infected *Aedes* mosquito [2]. Flaviviruses primarily infect skin Langerhan cells and dendritic cells, which travel to the lymph nodes, and from there, the virus is disseminated throughout the body [3]. DENV infection results in a broad spectrum of symptoms, ranging from dengue fever (DF), a self-limiting illness, to severe dengue with plasma leakage, severe bleeding, or organ impairment [4].

With 3 billion people living in regions of virus transmission and the increased risk of viral transmission to travelers to endemic countries, the development of a vaccine against DENV has been an ongoing challenge since its first isolation in 1943 [5]. While Dengvaxia licensure in 2016 has been a major advance in dengue vaccine development, limitations around its efficacy have required the ongoing development of alternative vaccines [6,7,8]. Intensive research has continued involving a range of vaccine approaches, with Takeda’s TAK-003 vaccine candidate seeking licensure for Europe and dengue-endemic countries, marking another important development for dengue vaccines [9].

Intradermal (ID) vaccination delivers antigen into the dermal layer of the skin, targeting the DCs, and eventually carries it to the draining lymph nodes, inducing a similar response to that which occurs when naturally infected with DENV via a mosquito bite [10,11]. However, the standard Mantoux ID vaccination is difficult to reproducibly perform without the aid of specialized needles (MicronJet and BD Soluvia Device) [10,12,13]. In recent years, advances in nano- and microfabrication processes have led to the development of specialized delivery technologies to overcome the technical challenges associated with ID vaccinations. Microarray patches, ranging from dissolving to solid microprojections, have been developed as alternative delivery platforms that offer a variety of benefits over the standard syringe and needle injection methods [14,15,16,17,18,19,20,21,22,23].

The HD-MAP is a 1 cm^2^ molded polymer patch, consisting of 5000 conical microprojections, each 250 µm in length [24]. The HD-MAP has been designed to target vaccine antigens to the dermal and epidermal layers of the skin, which are rich in antigen-presenting cells. To overcome the viscoelastic property of the skin, the HD-MAP is applied dynamically using a custom spring-loaded applicator. As a function of this application process, localized cell death is caused by the microprojections penetrating the skin, releasing damage-associated molecular patterns (DAMPs). The colocalization of the DAMPs with the delivered vaccine results in enhanced immune responses [8,25]. In comparative dose-matched studies with standard injection methods using mouse models and in Phase I clinical trials, the HD-MAP has been shown to elicit potent antibody responses using a fraction of a dose for a wide range of vaccines [17,24,26,27]. One of the unique features of the HD-MAP is the dry coating of the vaccine onto the surface of the microprojections. As a function of this drying, vaccines can be stabilized for extended periods with reduced dependence on the cold chain [26,28,29,30,31]. However, given the dry-coating process, there is a need to ensure the stability of the vaccine in a solid state upon dry down through careful selection and screening of excipients.

Our group has recently demonstrated the effective delivery of a novel chimeric Dengue virus vaccine candidate, BinJ/DENV2-prME (bDENV2), by the HD-MAP. The targeted delivery of an unadjuvanted, single dose of the vaccine to the skin raised potent immune responses that afforded complete protection to DENV challenge in the AG129 dengue mouse model when compared to the ID vaccinated group [27]. While these results were promising, retaining quaternary epitopes upon storage in their dry state was challenging. Using bDENV2, we explored 26 different classes and types of generally regarded as safe (GRAS) pharmaceutical excipients in various concentrations and identified those that would mitigate vaccine loss during the drying process and storage. The final candidate formulation with human serum albumin (HSA) maintained close to 100% recovery after 6 months of storage at 4 °C.

## 2. Materials and Methods

### 2.1. Animal Ethics

Animal experiments were approved by the University of Queensland (UQ) animal ethics committee (AEC no.: SCMB/AIBN/322/19/NHMRC) and performed in accordance with the National Health and Medical Research Council guidelines. Animals were maintained under specific pathogen-free conditions in the UQ Biological Resources animal housing facility.

### 2.2. Cell Lines

Vero cells (African green monkey kidney) were maintained at 37 °C with 5% CO_2_ in OPTI-MEM (Gibco, Darmstadt, Germany) cell culture medium, containing 3% fetal bovine serum (FBS, Bovogen). C6/36 (*Aedes albopictus*) cells were grown at 28 °C in Roswell Memorial Park Institute 1640 (RPMI, Gibco) medium, supplemented with 10% FBS, 100 U/mL penicillin (Gibco), and 100 μg/mL streptomycin (Gibco).

### 2.3. bDENV2 Chimeric Virus Propagation and Purification

The bDENV2 chimeras were generated in C6/36 cells as previously described by Choo et al. (2021) [27].

### 2.4. C8-Antigen-Capture Enzyme-Linked Immunosorbent Assay (ELISA)

The C8-antigen-capture ELISA used to analyze the reconstituted samples was performed as previously described [27]. Standard curves were generated by titrating 1 µg/mL of bDENV2 twofold across the plate. Fifty microliters/well of the reconstituted samples and the standard curve were added to the ELISA plate and incubated at 37 °C for one hour. Fifty microliters of humanized 513 DENV antibody [32] (h513), conjugated with horse-radish peroxidase (HRP), was incubated for one hour at 37 °C. Following incubation, 6× PBS-T washes were performed, and plates were visualized by adding tetramethylbenzidine-w (TMBW, BioFX, Eden Praire, MN, USA). The reaction was stopped by the addition of 1 M phosphoric acid, and the plate was read at 450 nm on a Varioskan LUX multimode plate reader (Thermo Fisher Scientific, Waltham, MA, USA).

As antigen recovery was judged by C8 binding, we developed a C8 antigen unit as a measure of vaccine potency. The C8 antigen unit was defined as the amount of vaccine in the C8 antigen conformation. The C8 antigen was calibrated against an internal reference standard of stock bDENV2 produced in house. One C8 antigen unit contained 31.25 ng of bDENV2.

### 2.5. Excipient Screening Studies to Identify Stabilizing Excipient for Dry Down and Storage

#### 2.5.1. Excipient Preparation

Stock excipients were prepared in sterile low-salt buffer (LSB, 20 mM Tris, and 75 mM NaCl, pH 7.4) to a final concentration of 5%. All solutions were sterile filtered through a 0.22 µm filter (Millipore, Burlington, MA, USA) and stored at −20 °C in single-use aliquots.

#### 2.5.2. bDENV2 Drying and Storage

To formulate bDENV2, 31.25 ng/mL of bDENV2 stock solution was mixed with the 5% stock excipients and LSB to obtain a final excipient concentration of 0.1%, 0.25%, 0.5%, 0.6%, 0.75%, or 1%. We dispensed 12 µL of this mixture onto the center of a 96-well plate (Thermo Fisher Scientific, Waltham, MA, USA), which was allowed to dry for 15 min under a 16 L/min nitrogen gas flow in a custom drying rig. Plates were sealed in foil bags and stored with desiccants.

#### 2.5.3. bDENV2 Reconstitution and Recovery for Analytical Testing

To reconstitute the dried bDENV2 samples, 100 µL of reconstitution buffer (1× KPL Milk diluent/Blocking solution concentrate (SeraCare) in PBS-T) were added to each well and pipetted up and down onto all quadrants and the middle of the well 10 times. The freshly reconstituted samples were immediately assayed by C8-antigen-capture ELISA as described in Section 2.4 without any storage.

#### 2.5.4. Recovery of bDENV2 Based on C8-Antigen-Capture ELISA

To determine the stability of bDENV2 after dry down and storage at different timepoints and temperatures, the standard curve in the C8-antigen-capture ELISA was used to interpolate and obtain the concentrations of bDENV2 (recovered and liquid stock control). The concentrations of the reconstituted and recovered dried vaccine were then normalized against the liquid stock control stored at 4 °C.

#### 2.5.5. Recovery of bDENV2 Based on 50% Tissue Culture Infectious Dose (TCID50) Assay

C6/36 cells were seeded in 96-well plates and grew to 80% confluency overnight at 28 °C. In separate round-bottom 96-well plates, reconstituted bDENV2 was serially diluted from 10^−1^ to 10^−10^. The diluted virus was added to the cells and incubated at 28 °C for 5 days. Following 5 day incubation, the medium was removed from the wells and plates were fixed with 80% acetone for 30 min at −20 °C. Fixative was removed, and plates were stained as described below.

#### 2.5.6. Immunoplaque Staining

Immunoplaque staining was performed as described by Choo et al. (2021) [27] with the changes stated below. Briefly, fixed plates were blocked for 1 h at room temperature. Following blocking, 1:1000 diluted anti-E mAb 4G2 antibody [33] was added for 1 h at 37 °C. Plates were washed four times in PBS-T, then 1:1000 diluted goat anti-mouse IR800 (Millennium) antibody was added for 1 h at 37 °C. The plates were washed again in PBS-T and dried before visualizing on an Odyssey Clx (LI-COR, Victoria, Australia). The Reed and Muench (1983) method [34] was used to calculate viral titer.

### 2.6. HD-MAPs Coating and Storage

HD-MAPs (1 cm^2^, 5000 projections/cm^2^ at 250 µM in length) were provided by Vaxxas Pty. Ltd., Brisbane, Australia. HD-MAPs were oxygen-plasma treated for 2 min before vaccine coating. The coating solution for each HD-MAP consisted of 3.15 µL of 5% methylcellulose, 3.15 µL of 5% human serum albumin (HSA, Sigma Aldrich, St. Louis. MO, USA), and 0.85 µL of 5.89 mg/mL purified bDENV2-prME and made up to 21 µL in LSB. The coating solution was dried onto microprojections using conditions previously described [27]. HD-MAPs that required storage were sealed with a desiccant and stored at 4 °C.

### 2.7. Scanning Electron Microscopy (SEM) of HD-MAPs

Fifteen nanometer platinum coatings were applied to HD-MAPs and imaged at a 45° angle using a Hitachi SU3500 at the Center of Microscopy and Microanalysis at UQ.

### 2.8. Evaluation of Stored bDENV2-Coated HD-MAP

Female BALB/c mice (6–8 weeks) were randomly split into four groups (*n* = 5) and vaccinated with bDENV2 HD-MAPs that had been coated immediately before application (bDENV2-immediate), coated then stored at 4 °C for 2 weeks before application (bDENV2-2 weeks), or coated and held for 1 month at 4 °C before application (bDENV2-1 month). HD-MAPs coated with excipients only were included as controls. Vaccine-/excipient-only-coated HD-MAPs were applied to the flank of the mice at a velocity of 20 m/s using a spring-loaded applicator. Tail bleeds were obtained on day 0 and cardiac bleeds on day 21 post-immunization. Blood samples were allowed to clot at 4 °C overnight, and the serum fraction was recovered by centrifugation at 10,000× *g* for 10 min at 4 °C. Serum was stored at −20 °C until further analysis.

### 2.9. DENV-Specific IgG ELISA

DENV-specific total IgG ELISA was performed as previously described by Choo et al. (2021) [27].

### 2.10. DENV Plaque Reduction Neutralization Tests (PRNTs)

PRNTs were performed as described by Choo et al. (2021) [27].

### 2.11. Optimization of Wear Time of HD-MAP after Application

To determine the impact of different wear times after application of HD-MAPs, female BALB/c mice (6–8 weeks old) were divided into eight groups (*n =* 8) for immunization by HD-MAP with bDENV2 with seven different wear times (0, 10, 30, 60, 90, 120, and 150 s) and an ID control vaccination group. Zero seconds (0 s) of wear time reflected removal of the patch immediately after delivery. Tail bleeds were obtained on day 0 and cardiac bleeds on day 21 post-immunization. The serum fraction was recovered as described above in Section 2.8.

### 2.12. Delivery Efficiencies of the Different Wear Times of HD-MAP

To determine the delivery efficiencies of the HD-MAP applied with different wear times, C8-antigen-capture ELISA was performed on the patches with the mentioned changes below. Briefly, remaining vaccines on coated HD-MAPs delivered to mice and coated HD-MAPs that remained undelivered were eluted off the patch by pipetting onto all four corners and the middle of the HD-MAP five times with blocking buffer. The HD-MAPs were then incubated at room temperature in an orbital shaker for 30 min. Fifty microliters/well of the eluted vaccines were added to the ELISA plate and incubated at 37 °C for one hour. The remaining C8 antigen was then measured using ELISA as described in Section 2.4. The amount of C8 antigen delivered was calculated by determining the difference between the C8 antigen recovered from coated HD-MAPs, pre- and post-application.

## 3. Results

### 3.1. Initial Excipient Screening Studies to Identify Stabilizing Excipients for Dry-Down and Storage

The retention of the native protein/virion structure during the phase changes between solid and liquid states is critical for inducing the correct immune response [35]. To minimize the loss of vaccine infectivity/potency/stability, a range of GRAS excipients approved by the Food and Drug administration were evaluated. As a surrogate for vaccine potency, we employed a virus-capture ELISA. The virus was captured by the quaternary epitope-specific monoclonal antibody C8 [36] and detected by monoclonal antibody 513 [32], both antibodies recognizing the envelope protein of all serotypes of DENV. Binding of a quaternary structure-specific antibody reflected the retention of an antigenically intact topography. As antigenic integrity was judged by C8 binding, we developed a C8 antigen unit as a measure of vaccine potency. This approach is modeled on the D antigen unit measure used for assessing inactivated poliovirus vaccine potency [37]. The C8 antigen unit was defined as the amount of vaccine displaying the C8 antigen conformation. The C8 antigen was calibrated against an internal reference standard of stock bDENV2 produced in house. One C8 antigen unit contained 31.25 ng of bDENV2.

**Figure 1:**
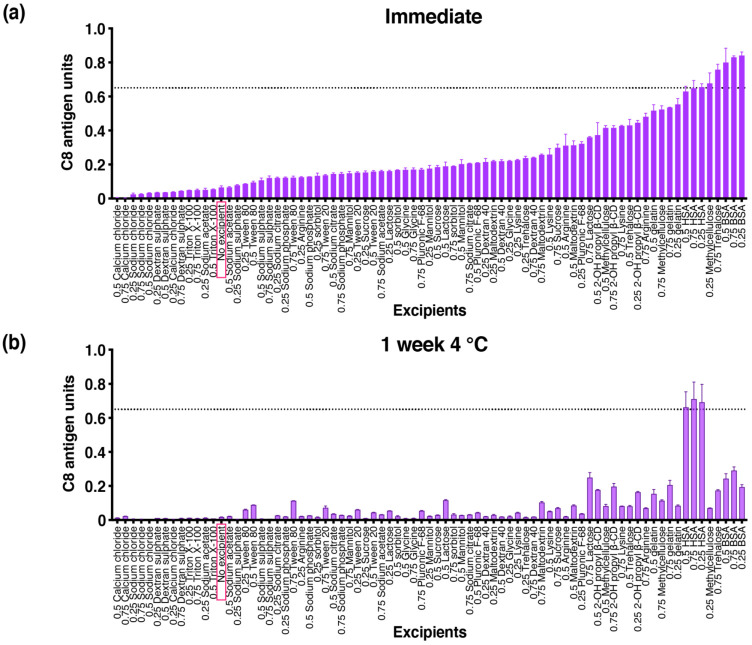
Effects of individual excipients on bDENV2 antigen recovery (**a**) upon immediate reconstitution after drying and (**b**) after storage for 7 days at 4 °C. Each condition is shown as a relative percentage recovery of bDENV2 antigen normalized to a bDENV2 stock liquid control (100%). Note: Excipients were prepared in a low-salt base buffer. Error bars represent SEM from triplicate experiments. The dotted line across the graph represents 65% recovery of bDENV2.

To investigate the stabilizing effects of the 26 GRAS excipients, samples were dried in their presence using a 96-well-plate drying system. The 26 different excipients were prepared in 3 concentrations (0.25%, 0.5%, 0.75%) and were individually mixed with 1 C8 antigen unit of vaccine. The test formulations were dried down in 96-well plates under a nitrogen gas stream. Plates were either immediately reconstituted (Figure 1a) to determine the amount of C8 antigen loss upon dry down or stored at 4 °C (Figure 1b), ambient temperature, or 37 °C (Figure S1) for 7 days.

Of the 79 formulations evaluated in the immediate timepoint, 43 formulations showed significantly improved C8 antigen retention when compared against the no-excipient control (bDENV2 dried down with LSB only, Figure 1a). Examples of the excipients that demonstrated improved dry-down stability identified in the initial screen were protein carriers (bovine serum albumin (BSA), has, and gelatin), sugars (methylcellulose, trehalose, lactose), amino acids (arginine and lysine) as well as 2-OH propyl-β cyclodextrin. After 7 days of storage at 4 °C, only 14 formulations were significantly higher when compared against the no-excipient control (Figure 1b). Like the immediate timepoint, stabilizing excipients identified were BSA, HSA, gelatin, lactose, and 2-OH propyl-β cyclodextrin. Interestingly, the percentage recovery of HSA at all three concentrations after 7 days of storage was observed to be higher than its immediate timepoint (Figure 1b). Based on this initial screen, and its ability to mitigate C8 antigen loss during both dry down and short-term storage, HSA was chosen as the lead excipient.

### 3.2. Optimizing Dry-Down and Reconstitution Buffers

**Figure 2:**
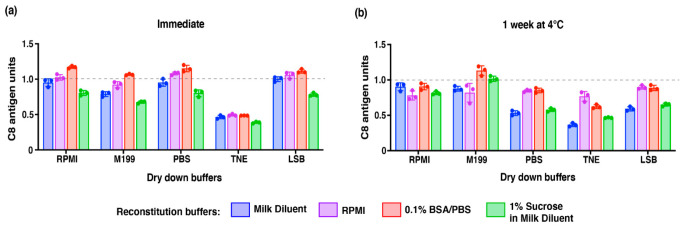
Optimization of drying and reconstitution buffers for 0.75% HSA/bDENV2. Briefly, 0.75% HSA/bDENV2 was dried on a 96-well plate using various buffers (RPMI, M199, PBS, TNE, and LSB) and reconstituted with different buffers (milk diluent, RPMI, 0.1% BSA/PBS, and 1% sucrose in milk serum) (**a**) immediately upon drying or (**b**) after 7 days of storage at 4 °C. Analysis was conducted using subtractive DENV E ELISA, and percentage recovery of bDENV2 antigen was normalized against a liquid stock control. Note: Error bars represent SEM from triplicate experiments. Dotted line across the graph represents 100% recovery of C8 antigen.

As previously identified by Wan et al. (2018) [31], losses of vaccine potency are not limited solely to the dry down and storage process but also occur due to some of the virus particles adsorbing onto the container upon reconstitution. To mitigate this loss, we looked to optimize dry-down and reconstitution buffers to maximize recovery of C8 antigen immediately and 7 days after drying at 4 °C. We tested 0.75% HSA/bDENV2 with commonly used buffers PBS and TNE (10 mM Tris, 120 mM NaCl, and 1 mM EDTA) and LSB; a buffer used previously for dry down on HD-MAPs [18,26,27]. Cell culture media such as RPMI and M199 was also selected for testing due to success in previous studies [31]. Following immediate reconstitution, the pairing of drying with LSB and reconstitution with milk serum in PBS-T resulted in the complete recovery of C8 antigen (Figure 2a). It was also observed that drying in TNE resulted in the poorest recovery (~0.5 C8 antigen units) regardless of reconstitution buffer used. Similarly, reconstituting in 1% sucrose in milk diluent in PBS-T gave the poorest recovery regardless of dry-down buffer (Figure 2a). Across all groups (except for TNE), it was observed that reconstituting with 0.1% BSA in PBS gave the highest recovery of C8 antigen (~1.1 C8 antigen units; Figure 2a). Upon recovery after a week’s storage at 4 °C, groups using salt-based drying buffers (PBS, TNE, and LSB) resulted in a greater loss of C8 antigen when compared to using cell culture media (Figure 2b). Reconstitution with 0.1% BSA/PBS yielded the highest recovery when pairing with M199 as the drying buffer (Figure 2b). Moving forward, all experiments performed in this study used M199 in the dry down and reconstitution with 0.1%BSA/PBS.

### 3.3. Long-Term Stability of bDENV2 in a Dried State

Following the identification of the lead stabilizing excipient and formulation conditions for the bDENV2 vaccine candidate, we proceeded to evaluate the long-term stabilizing effects HSA imparts upon bDENV2. One C8 antigen unit of bDENV2 was formulated in HSA (0.1%, 0.25%, 0.5%, 0.6%, 0.75%, and 1%) in M199 in 96-well plates. bDENV2 formulated without HSA, in M199 only, was included as the control. Dried plates were stored with desiccants from 1 day to 6 months at 4 °C.

**Figure 3:**
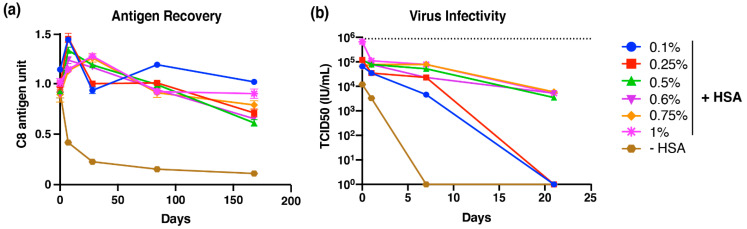
Long-term stability of bDENV2 formulated with different concentrations of HSA in M199 at 4 °C. (**a**) Dried bDENV2 stored at 4 °C with desiccants from 1 week to 6 months was subjected to C8-antigen-capture ELISA to analyze antigen recovery. Each condition is shown as a relative percentage recovery of bDENV2 antigen normalized to a bDENV2 stock liquid control (100%). (**b**) Dried bDENV2 stored at 4 °C with desiccants from 1 to 3 weeks was subjected to TCID50 for analysis of viral infectivity. Dotted lines represent titer (10^6^ TCID50 IU/mL) of viable bDENV2 prior to storage. Error bars represents SEM from triplicate experiments.

Upon immediate dry down and reconstitution, 0.9–1.1 C8 antigen units were recovered for all HSA concentrations and the control group without HSA (Figure 3a), suggesting that minimal losses in antigenic integrity occur during the drying process. However, in the absence of HSA, a dramatic loss in C8 antigen recovery was observed after even short storage periods. Minimal loss in C8 antigen unit recovery was observed for all concentrations of HSA tested, even out to 6 months of storage. Given the trend for C8 antigen unit loss for the other HSA concentrations tested, it is possible that even at 1% HSA, further losses may be observed over time.

As the chimeric virus can replicate in mosquito cells, we next sought to determine whether we were able to recover infectious virus particles as an additional marker of stability. Using the lead stabilizing excipient, we evaluated the infectivity of reconstituted bDENV2 overtime via a TCID50 infectivity assay. Losses in virus infectivity were observed during the drying process when bDENV2 was formulated in 0.1–0.75% HSA (Figure 3b). However, no loss on dry down was observed when bDENV2 was formulated in 1% HSA. We observed approximately 0.5 to 1 log loss in virus infectivity for all tested conditions following one day of storage. In the absence of HSA, bDENV2 lost all virus infectivity after 7 days of storage (Figure 3b). Following 21 days of storage, bDENV2 formulated in 0.1% and 0.25% HSA lost virus infectivity, while bDENV2 formulated in ≥0.5% HSA retained infectivity to at least 10^4^ TCID50/mL (Figure 3b). Based on the recovery of antigenic integrity and virus infectivity over these extended periods, 1% HSA/bDENV2 was chosen as the lead formulation.

### 3.4. Store and Patch Study: The Delivery of Stable bDENV2 via HD-MAP

**Figure 4:**
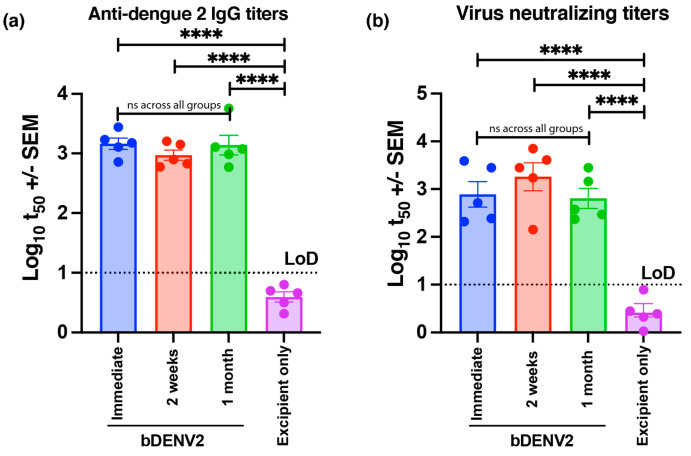
Store and patch study: The delivery of stable bDENV2 via HD-MAP. bDENV2 was coated on the HD-MAP and stored for 2 weeks or a month at 4 °C before being used to vaccinate female BALB/c mice. HD-MAPs freshly coated with bDENV2 or excipient only were included as controls for this study. (**a**) The IgG response for sera taken 21 days post-vaccination was determined, and mid-point (t50) antibody titers were plotted. (**b**) Neutralizing antibody titers were determined for sera obtained 21 days post-vaccination against DENV2 ET00. Note: Each symbol represents a single mouse. The line indicates mean antibody titers with bars showing ± SEM. **** *p* ≤ 0.0001 assessed by one-way analysis of variance (ANOVA, α-level 0.05).

The recovery of bDENV2 antigen and infectious particles after dry-down storage in microtiter plates has previously been shown to be a valid surrogate measure of storage on the HD-MAP [31]. However, the stability of a vaccine over long-term storage is not solely defined by the recovery of antigen or infectious particles but the functional output of its ability to retain release potency to generate an appropriate immune response capable of neutralizing the infectious virus. To investigate vaccine potency retention following storage, bDENV2-coated HD-MAPs were stored for 2 weeks and a month at 4 °C before vaccination of female BALB/c mice. HD-MAPs, coated and immediately used to vaccinate mice, were the positive controls. A smooth and even coating of bDENV2 was observed on the microprojections of the HD-MAP (Figure S2a). HD-MAPs post-application were also imaged by scanning electron microscopy, revealing no difference between the removal of vaccine from the tip of the microprojections between different storage time points (Figure S2b,c). When evaluated for total DENV IgG titers, no significant differences were observed between any of the HD-MAP-vaccinated groups regardless of storage period (Figure 4a). Similarly, neutralizing titers were observed to have no significant differences between vaccinated groups (*p <* 0.0001), indicating complete retention of vaccine potency (Figure 4b).

### 3.5. Optimization of HD-MAP Wear Time

**Figure 5:**
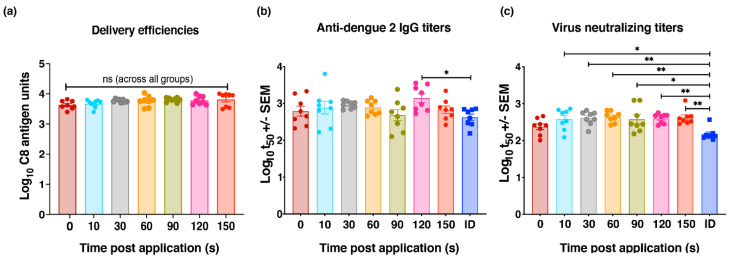
Optimization of HD-MAP wear time. (**a**) bDENV2 delivered by HD-MAP after different wear times post-application. C8 antigen capture ELISA was performed to analyze the amount of vaccine delivered. The bar graph represents the mean of bDENV2 (*n* = 8) delivered by HD-MAPs across different wear times with error bars indicating SEM. (**b**) The IgG response for sera obtained after final vaccination was determined with mid-point (t50) antibody titers plotted. (**c**) Neutralizing antibody titers were determined for sera obtained 21 days post-vaccination against DENV2 ET00. Note: Each symbol represents a single mouse. The line indicates mean antibody titers with bars showing ± SEM. ** *p* ≤ 0.002, * *p* ≤ 0.03 assessed by one-way analysis of variance (ANOVA, α-level 0.05).

As standard practice, previous studies have used a somewhat arbitrary 2 min HD-MAP wear time to allow the deposition of vaccine antigen into the skin [15,16,17,18,19,25,26,27]. Given that each vaccine has a unique formulation, we reasoned that the kinetics of vaccine delivery is likely influenced by formulation. To determine the shortest amount of wear time required to deliver the desired amount of vaccine to the skin, HD-MAPs were coated with bDENV2 and applied to the mouse flank and then left in place for 0, 10, 30, 60, 90, 120, and 150 s. Delivery efficiencies were determined, revealing that there was no significant difference between any wear times tested (Figure 5a), including the HD-MAPs removed immediately after application. Following vaccination, IgG-antibody- and virus-neutralizing titers were analyzed with collected sera. Again, no significant difference was observed in the antibody titers with increasing wear times (Figure 5b), although sera from mice receiving the vaccine via ID injection showed significantly lower antibody titers than the 120 s wear time group (*p =* 0.05; Figure 5b). Similarly, virus-neutralizing titers mirrored the IgG titers, with no significant differences between wear times (Figure 5c). However, when compared to the ID control group, neutralizing titers were observed to be significantly higher in groups with greater than 10 s of wear time (10 s: *p =* 0.0131; 30 s: *p =* 0.0053; 60 s: *p =* 0.0022; 90 s: *p =* 0.0134; 120 s: *p =* 0.0073; and 150 s: *p =* 0.0035; Figure 5c). Collectively, these results suggest wear times may need to be less than previously thought and should be empirically determined for each new vaccine formulation.

## 4. Discussion

In the past decade, vaccine delivery platforms using microprojection array patches have been developed as an alternative to the traditional needle and syringe injection method. As the HD-MAP uses a self-disabling applicator to vaccinate individuals, this vaccine delivery platform avoids many of the drawbacks of the needle and syringe such as vaccine reconstitution, sharp waste, or requiring highly trained medical staff to administer the vaccine. Here, we have successfully identified a candidate formulation that confers antigenic stability to a live chimeric flavivirus vaccine candidate when dried on the HD-MAP for at least 6 months.

One of the challenges of the HD-MAP delivery platform is formulating a vaccine product that is resistant to the stressors associated with phase changes during the drying process. Using an approach similar to that described by Wan et al. (2018) [31], we screened 26 GRAS excipients in 78 formulations to identify excipients that protected the vaccine during the drying process and stabilized the vaccine over time. Initial screening of individual excipients identified sugars (trehalose and methylcellulose) and carrier proteins as candidates that protected the C8 quaternary antigen content of the chimeric virus from the drying process. Consistent with a previous study, sugar-based formulations maintained the viral potency of DENV when dried on microarray patches [38]. In addition to the sugars, carrier proteins such as BSA, HSA, and gelatin were also beneficial during the dry-down process. However, upon storage for a week at 4 °C, bDENV2 formulated in trehalose, methylcellulose, BSA, or gelatin showed a significant loss of C8 antigen content. When formulated in HSA, bDENV2 maintained C8 antigen potency as observed from data obtained immediately after the drying process. Others have observed similar effects, with HSA acting as both a carrier protein and an effective vaccine stabilizer [39,40,41,42].

Following refinement of the initial excipient screenings, 1% HSA was identified as the optimal stabilizing excipient for bDENV2. The use of HSA as an excipient in vaccine formulations has been explored widely as it is commonly used as a stabilizing agent for proteins and enzymes in pharmaceutical settings. As HSA is capable of acting as a cryoprotectant for proteins during lyophilization [43], it has been used as an excipient in various vaccine formulations, such as DENVax [42], rVSV-ZEBOV [39], M-M-R^®^ II [41], and Varilrix^TM^ [40]. Using 1% HSA, >90% of C8 antigen content was retained after 6 months of storage at 4 °C. While the antigenic content of the virus was retained, 99% of viral infectivity was lost after 3 weeks of storage. Despite the loss of virus infectivity, antibodies elicited from immunized animals were similar at all time points tested. These results confirm that the potent virus-neutralizing antibodies produced were a function of the immune response to the virion itself and not related to de novo translation of viral proteins or limited RNA replication. This is supported by our previous work demonstrating the absence of RNA replication or de novo synthesis in mammalian cells [27].

The major advantage of the intramuscular injection delivered by the hypodermic needle and syringe is the almost instantaneous speed at which vaccine doses are administered. Microarray patch technologies rely on the dissolution of the vaccine into the skin upon application. As such, reported wear times range from 2 to 20 min to allow for vaccine deposition into the skin. Extended wear times are not compatible with efficient immunization in mass vaccination settings. To determine the shortest wear time required to facilitate vaccine deposition into the skin and maximal immune response, we investigated delivery efficiencies and corresponding immune responses as a function of time.

Interestingly, immediate removal of the HD-MAP following application resulted in the same vaccine delivery into the skin as our standard 2 min application time. While excipients present in the vaccine formulation may likely influence vaccine transfer efficiency, our results suggest that the main contributor to vaccine removal from the projections is mechanical stripping upon entry or exit from the skin rather than the previously proposed vaccine diffusion [44]. This observation is supported by the fact that total anti-DENV2 IgG and neutralizing antibody titers generated were not influenced by the different HD-MAP wear times. While these are promising results, a limitation of this study is the difference in skin thickness of mice compared to human skin. Although we anticipate similar findings, additional investigations will be required to confirm the dynamics of vaccine deposition in human skin.

## 5. Conclusions

Enveloped viruses such as DENV are inherently unstable and not readily amenable to storage in a dried format. Through extensive screening, we identified 1% HSA as a potent stabilizer of bDENV2, protecting the antigenic content of the bDENV2 virion for 6 months in a dried format. We showed that mice immunized with a stored bDENV2-coated HD-MAP still produced potent virus-neutralizing antibody responses to confirm the relationship between stability and vaccine potency. This result, coupled with ultra-short wear time and ease of use, makes the HD-MAP technology an attractive alternative to traditional needle and syringe injection delivery in mass vaccination settings.

## Data Availability

The authors declare that all data supporting the findings of this study are available within the paper and its Supplementary Materials.

## Supplementary Materials

The following are available online at https://www.mdpi.com/article/10.3390/vaccines9111301/s1, Figure S1: Effects of individual excipients on bDENV2 antigen recovery after storage for 7 days at 25 °C or at 37 °C, Figure S2: SEM images of coated HD-MAP pre- and post-application.
