# Supplementary material for: Developing a Stabilizing Formulation of a Live Chimeric Dengue Virus Vaccine Dry Coated on a High-Density Microarray Patch
Source: Vaccines (Basel). 2021 Nov 9;9(11):1301. doi: 10.3390/vaccines9111301 (PMC8625757; doi:10.3390/vaccines9111301)
Supplement: Supplementary file 1 [file vaccines-09-01301-s001.zip › vaccines-1436826-supplementary.pdf]

(a)

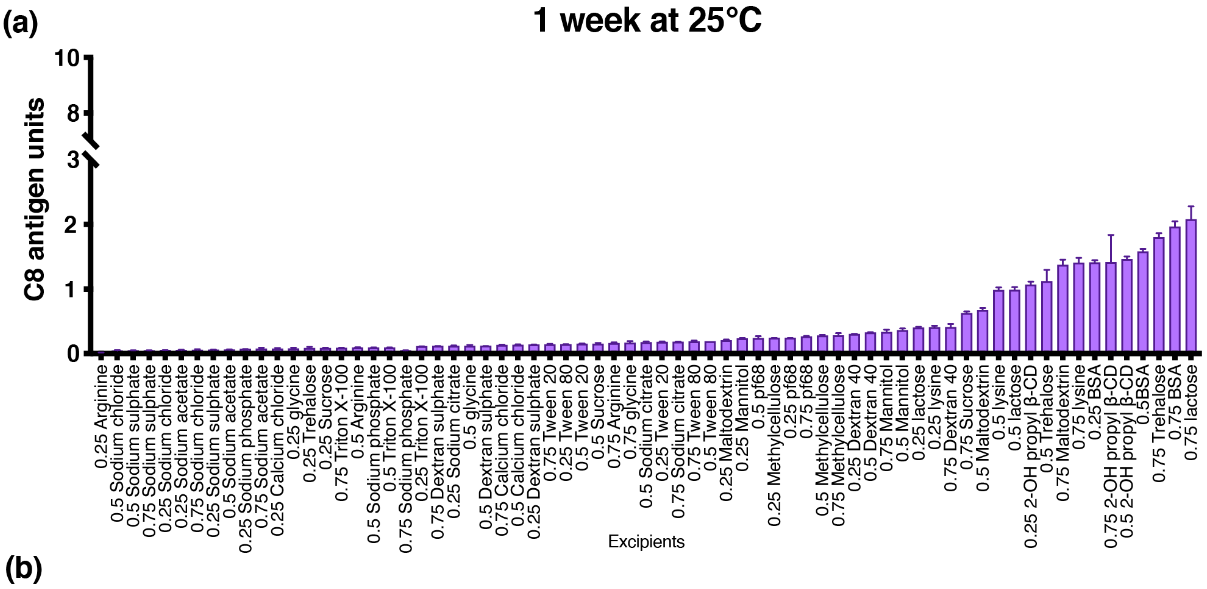

(b)

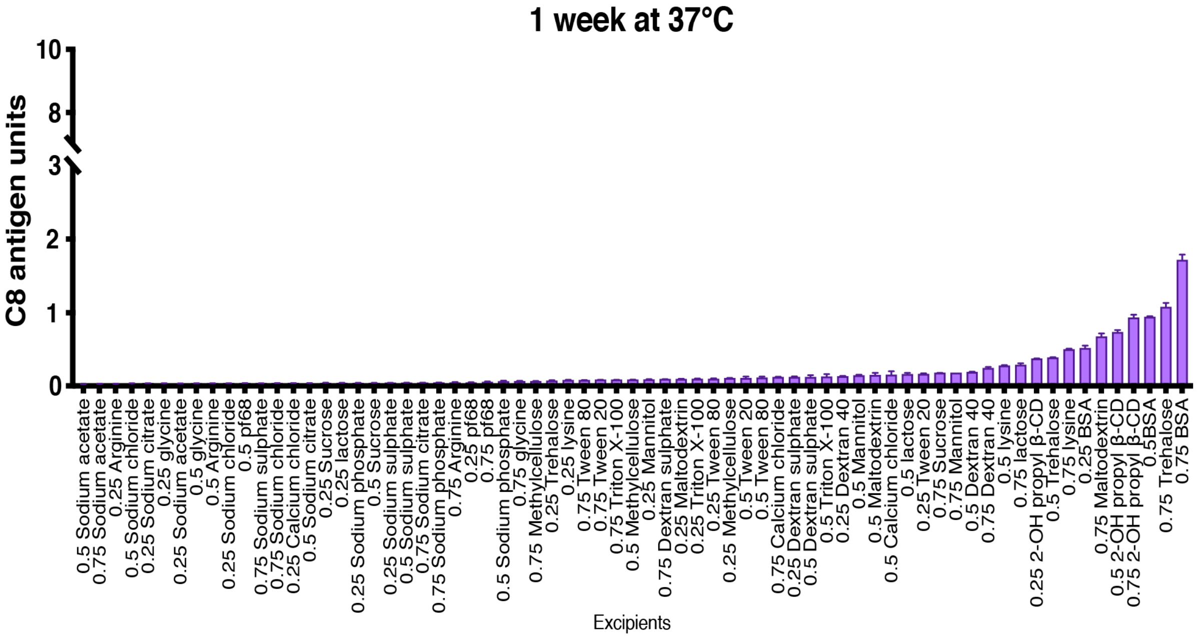

Figure S1. Effects of individual excipients on bDENV2 antigen recovery (**a**) after storage for 7 days at 25 °C and (**b**) after storage for 7 days at 37 °C. Each condition is shown as a relative percentage recovery of bDENV2 antigen normalized to a bDENV2 stock liquid control (100%). Note: Excipients were prepared in a low salt base buffer. Error bars represent SEM from triplicate experiments.

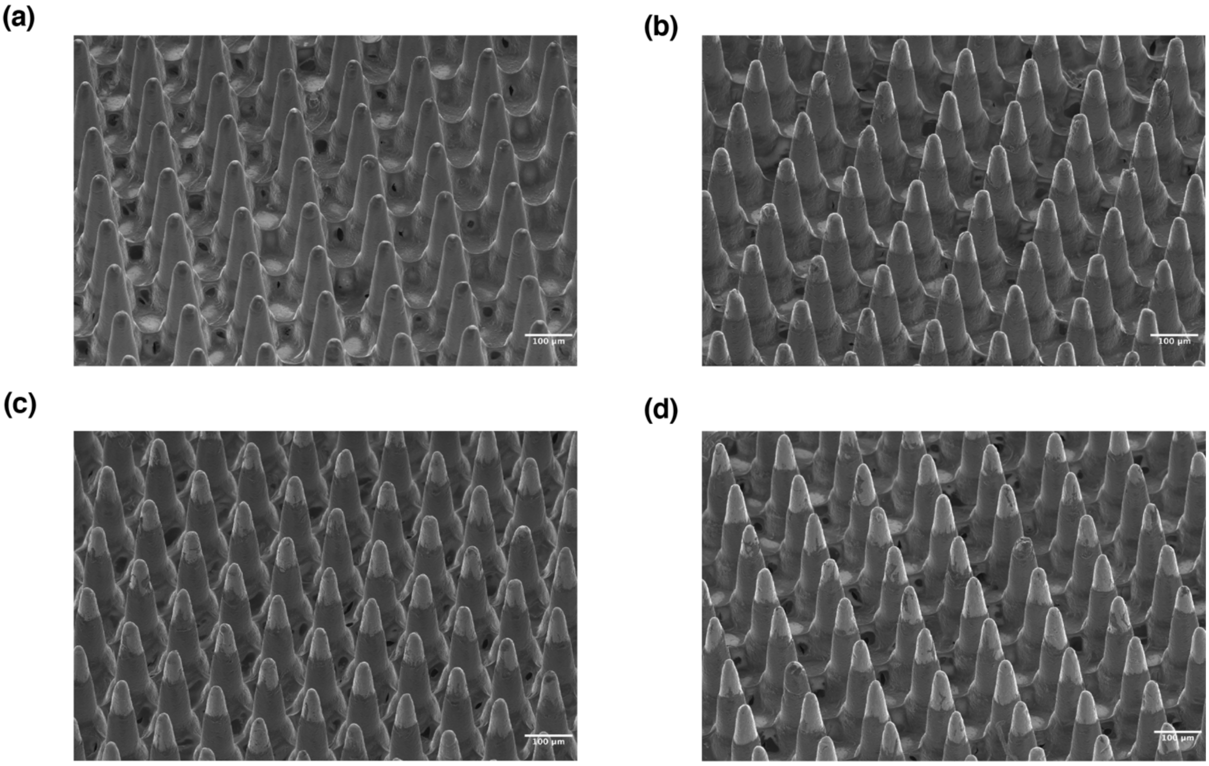

Figure S2. SEM images of (**a**) HD-MAP coated with bDENV2, (**b**) bDENV2-immediate HD-MAP post application, (**c**) bDENV2-2weeks HD-MAP post application, and (**d**) bDENV2-1month HD-MAP post application.
